# Technology-Intensified Diabetes Education Study (TIDES) in African Americans with type 2 diabetes: study protocol for a randomized controlled trial

**DOI:** 10.1186/1745-6215-15-460

**Published:** 2014-11-25

**Authors:** Joni S Williams, Cheryl P Lynch, Rebecca G Knapp, Leonard E Egede

**Affiliations:** Division of General Internal Medicine & Geriatrics, Medical University of South Carolina, 135 Rutledge Avenue, Charleston, SC 29425 USA; Center for Health Disparities Research, Medical University of South Carolina, 135 Rutledge Avenue, Charleston, SC 29425 USA; Health Equity and Rural Outreach Innovation Center, Ralph H Johnson VAMC, 109 Bee Street, Charleston, SC 29401 USA; Department of Public Health Sciences, Medical University of South Carolina, 135 Cannon Street, Charleston, SC 29425 USA

**Keywords:** Diabetes, Education, Skills training, Technology, Telehealth, Telemedicine

## Abstract

**Background:**

Compared to American Whites, African Americans have a higher prevalence of type 2 diabetes mellitus (T2DM), experiencing poorer metabolic control and greater risks for complications and death. Patient-level factors, such as diabetes knowledge, self-management skills, empowerment, and perceived control, account for >90% of the variance observed in outcomes between these racial groups. There is strong evidence that self-management interventions that include telephone-delivered diabetes education and skills training are effective at improving metabolic control in diabetes. Web-based home telemonitoring systems in conjunction with active care management are also effective ways to lower glycosylated hemoglobin A1c values when compared to standard care, and provide feedback to patients; however, there are no studies in African Americans with poorly controlled T2DM that examine the use of technology-based feedback to tailor or augment diabetes education and skills training. This study provides a unique opportunity to address this gap in the literature.

**Methods:**

We describe an ongoing 4-year randomized clinical trial, which will test the efficacy of a technology-intensified diabetes education and skills training (TIDES) intervention in African Americans with poorly controlled T2DM. Two hundred male and female AfricanAmerican participants, 21 years of age or older and with a glycosylated hemoglobin A1c level ≥8%, will be randomized into one of two groups for 12 weeks of telephone interventions: (1) TIDES intervention group or (2) a usual-care group. Participants will be followed for 12 months to ascertain the effect of the interventions on glycemic control. Our primary hypothesis is that, among African Americans with poorly controlled T2DM, patients randomized to the TIDES intervention will have significantly greater reduction in glycosylated hemoglobin A1c at 12 months of follow-up compared to the usual-care group.

**Discussion:**

Results from this study will add to the current literature examining how best to deliver diabetes education and skills training and provide important insight into effective strategies to improve metabolic control and hence reduce diabetes complications and mortality rates in African Americans with poorly controlled T2DM.

**Trial registration:**

This study was registered with the National Institutes of Health Clinical Trials Registry on 13 March 2014 (ClinicalTrials.gov identifier# NCT02088658).

## Background

Diabetes mellitus affects approximately 29 million adults in the United States [1]. African Americans (AAs) have a higher prevalence of type 2 diabetes mellitus (T2DM), poorer metabolic control and greater risk for complications and death compared to White Americans [2]. There is strong evidence that self-management interventions that include diabetes education and skills training are effective at improving metabolic control in diabetes [3]. Recent findings indicate that patients with diabetes, especially ethnic minority patients, prefer telephone-delivered diabetes education to group visits or internet-based education [4]. Multiple randomized controlled trials have documented the effectiveness of telephone-delivered self-care interventions in T2DM [5–8]. Preliminary data from our group suggest that a culturally tailored telephone-delivered diabetes education and skills training intervention [9] is an effective strategy to improve metabolic control in AA patients with T2DM.

Use of telephone contact, video-conferencing, personal digital assistants and web-based systems offer new opportunities to bridge the gap in support for patients with diabetes between face-to-face visits with their healthcare providers. Three large-scale randomized controlled studies have shown that home telemonitoring in conjunction with active care management resulted in lower glycosylated hemoglobin A1c (HbA1c) values at follow-up when compared to standard care [8, 10, 11].

**Figure 1:**
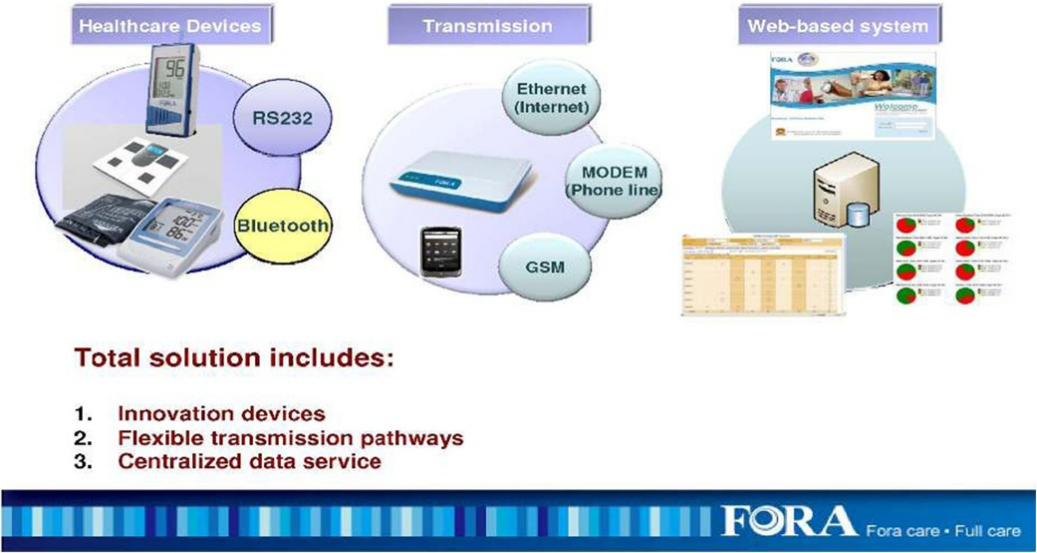
**The FORA TeleHealth System.** GSM, Global System for Mobile communications.

**Figure 2:**
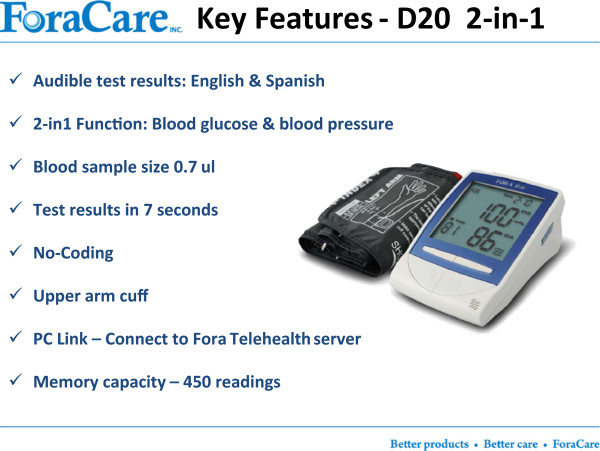
**The FORA two-in-one blood glucose and blood pressure device (D20).**

**Figure 3:**
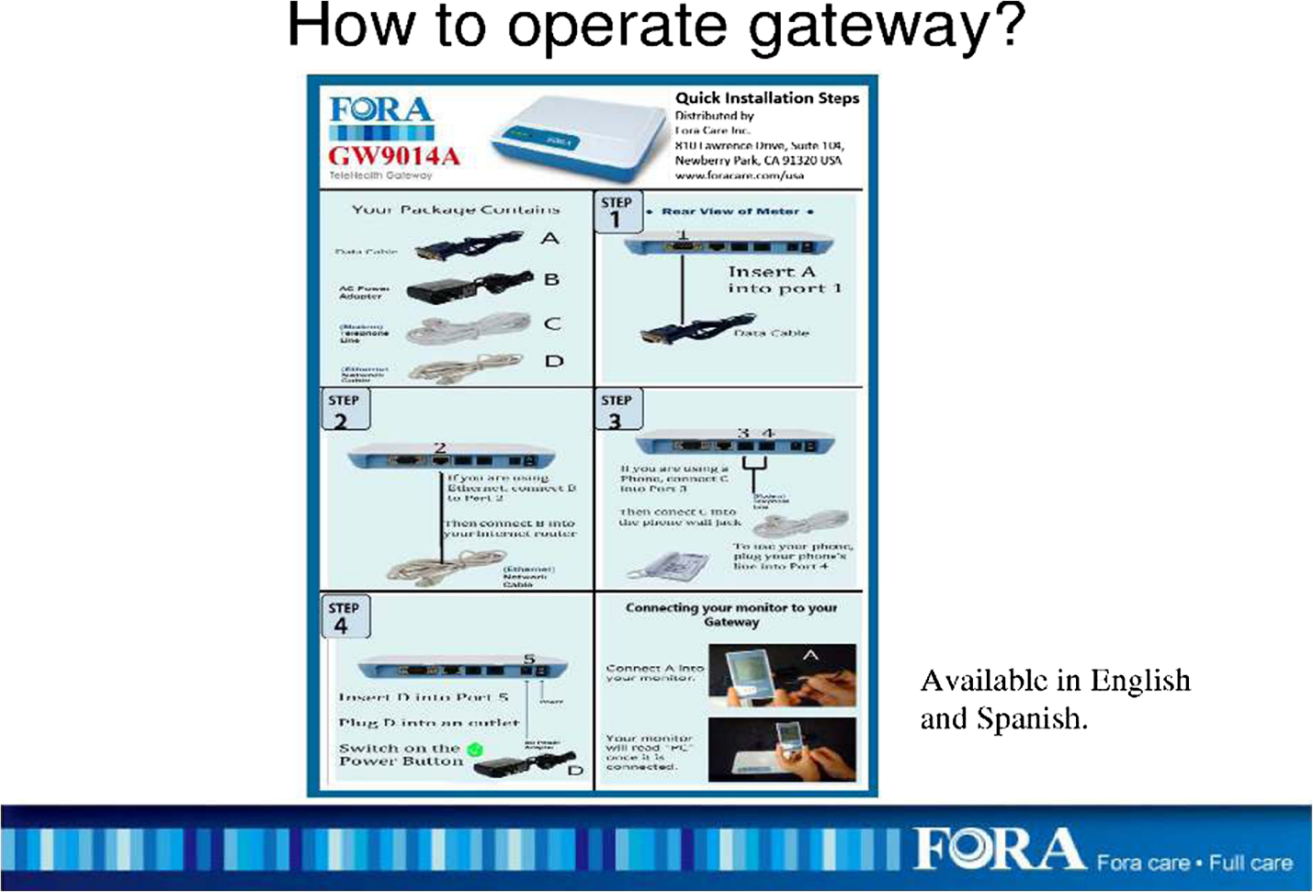
**Installation instructions for the FORA TeleHealth gateway.**

The FORA system (Fora Care Inc. 893 Patriot Drive, Suite D, Moorpark, CA 93021, USA) is an inexpensive, off-the-shelf, state-of-the-art technology whereby a person/caregiver and a provider can communicate accurately on data needed for self-management of diabetes. The system is comprised of an easy to operate two-in-one blood glucose and blood pressure monitor (Figures 1 and 2) that uploads results to a secure website via a modem. The system provides an easy-to-use operation for users to accomplish two important tests. The measured results can be uploaded to a personal computer or web-based software by using a RS232 cable via a communication device to connect the FORA glucose monitor and web-based FORA TeleHealth System using a phone modem (Figure 3). The provider (in this case a nurse case manager (NCM) or diabetes educator (DE)) can review the glucose and blood pressure readings weekly and use the data to tailor diabetes education and self-care skills training using motivational enhancement techniques to achieve optimal metabolic control.

This paper describes the rationale, study aims and objectives, and research design and methods of an ongoing 4-year, randomized clinical trial to test the efficacy of a technology-intensified diabetes education/skills training (TIDES) intervention in AAs with poorly controlled T2DM. The long-term goal of the project is to identify effective strategies to improve metabolic control and hence reduce diabetes complication and mortality rates in AAs with T2DM.

### Rationale

AAs have a higher prevalence of T2DM, poorer metabolic control (that is, poorer blood glucose, blood pressure, and lipid control), and greater risk for complications and death compared to White Americans [2]. HbA1c is the primary marker for glycemic control and is a strong independent predictor of developing complications and increasing mortality in T2DM [12]. Key self-care behaviors that influence glycemic control (and HbA1c) include diet, physical activity, self-monitoring of blood glucose and medication adherence [12].

No large randomized controlled trial has tested whether providing feedback to patients using novel technology such as the FORA system leads to improvements in glycemic and blood pressure control or whether using the feedback to tailor and augment diabetes education and skills training are effective at improving metabolic control. More importantly, there are no studies in AAs with poorly controlled T2DM that examine the use of technology-based feedback to tailor and/or augment diabetes education and skills training (that is, TIDES). This study provides a unique opportunity to address this gap in the literature.

### Study aim and objectives

The primary aim of this study is to test the efficacy of a TIDES intervention using the FORA two-in-one and TeleHealth System for diabetes in improving HbA1c levels in AAs with poorly controlled T2DM. The primary outcome is HbA1c at 12 months post-randomization, while the secondary outcomes are blood pressure (BP) control, quality of life (QoL), and resource utilization/cost at 12 months post-randomization.

## Methods

The study, which was funded in May 2013 and has an anticipated closure date of April 2017, is an ongoing, 4-year, two-group randomized controlled trial with randomization of individual participants, blinded outcomes assessments at baseline, 3, 6, 9, and 12 months, and concurrent economic evaluation.

### Location and setting

The study sites for this study include the general internal medicine, endocrine, family medicine, and community care clinics affiliated with the Medical University of South Carolina in Charleston, SC, USA.

### Ethics and trial registration

The study is funded by grant R01DK098529 from the National Institute of Diabetes and Digestive and Kidney Diseases. The trial is approved by the Institutional Review Board (IRB) of the Medical University of South Carolina (Pro#00015064; IRB approval date: 17 January 2012). The trial is registered (registration date, 13 March 2014) on the United States National Institutes of Health Clinical Trials Registry (ClinicalTrials.gov identifier# NCT02088658), available online at: http://clinicaltrials.gov/ct2/show/NCT02088658.

### Trial population and recruitment

A total of 200 AAs with T2DM will be randomized to one of two groups: 1) TIDES intervention group, which consists of telephone-delivered diabetes knowledge/information and skills; and 2) usual care.

We use two complementary approaches to identify eligible study participants. The first method consists of systematic identification of AA patients with T2DM. After obtaining approval for a partial waiver of the Health Insurance Portability and Accountability Act from our local IRB, we use clinic billing records over the previous 12-month period to identify AA patients with International Classification of Diseases, 9th revision, codes consistent with a diagnosis of T2DM. The physicians of eligible patients are notified of their patients’ potential eligibility and asked permission to enroll their patients in this study. After consent is obtained from the physicians, letters of invitation on clinic letterhead signed by the patient’s physician are mailed to patients from the study clinics. The letter provides information about the study, explains the study requirements, and clarifies that only patients who meet certain criteria are eligible to participate in the study. The letter includes an addressed and stamped postcard that patients can mail back to indicate interest or lack of interest in participating in the study. In addition, the letter provides a telephone number that interested patients can call to receive detailed information about the study. In the letter, patients are also informed that they will receive a follow-up call in 2 weeks unless they mail back the postcard or call to decline being contacted. Patients who mail back the postcard and express interest or call the provided telephone number receive detailed information about the study. Patients who agree to participate are asked to provide written consent and are scheduled for the initial screening assessment.

The second method consists of referrals from physicians, other clinic staff such as nurses, or patients themselves in response to recruitment flyers for the study. The Principal Investigator shares the goals of the study and inclusion/exclusion criteria with physicians and clinic staff during clinic administrative meetings. Physicians and clinic staff are asked to refer appropriate patients to the study research assistants. In addition, IRB-approved recruitment flyers are posted in prominent locations in the study clinics.

Regardless of the recruitment pathway, research staff members obtain written informed consent, complete screening for eligibility, and assure that participants meet criteria for inclusion and participation in the study. The procedure and risks are explained to the patients and the consent form signed as per standard clinical practice. Participants who meet eligibility criteria then complete the remainder of the assessment battery.

### Randomization

All participants are randomly assigned to one of the two study arms (n = 100 per arm). The study coordinator verifies all eligibility criteria prior to randomization. A permuted block randomization method is used to assign participants to one of the two groups: (a) TIDES intervention; and (b) usual care. Block size will vary to minimize the likelihood that the blind will be broken. The randomization is stratified by baseline HbA1c levels (8 to 10% versus >10%). Using Research Electronic Data Capture, research assistants collect eligibility information and enter the information into the study database via the secured study website [13]. Once eligibility is confirmed, the computer generates the intervention assignment based on the pre-programmed randomization scheme. All participants who are randomized are entered into the study database and analyzed according to Consolidated Standards of Reporting Trials guidelines [14].

### Intervention and control groups

There is one active treatment group (combined telephone-delivered diabetes knowledge/information and telephone-delivered motivation/behavioral skills training) and a usual-care group.

#### Description of the technology-intensified diabetes education/skills training intervention

The intervention is based on the information-motivation-behavioral skills model [15] and provides information, motivation, and behavioral skills training (using motivational enhancement techniques). Patients are assigned the FORA two-in-one TeleHealth System and provided with glucose test strips to allow testing at least once a day. They are asked to perform glucose testing and blood pressure measurement using the FORA system once daily. They are asked to upload the measurements daily as soon as possible after the test is performed. The NCMs or DEs have access to a secure server to which the uploaded measurements are stored in real time. The glucose and BP readings are used to tailor and reinforce behavior change during weekly telephone-delivered diabetes education sessions. The TIDES intervention in essence facilitates increased frequency of self-monitoring and titration of skills training (that is, glucose testing, diet, physical activity, and medication adherence) in response to test values to optimize diabetes and blood pressure control. Participants randomized to this group receive: 1) the FORA system for self-monitoring; 2) weekly telephone-delivered diabetes education/skills training; 3) patient activation (list of five questions to ask their provider at every visit and training on how to ask the questions); and 4) patient empowerment (diabetes responsibility contracts, personal goals, and flow charts for patients to record laboratory results/medications and training on how to use the empowerment tools). The intervention is delivered by telephone once a week for 12 weeks with each session lasting approximately 30 minutes.

#### Description of the usual-care group

Apart from study visits, patients randomized to the usual-care group are followed by their primary-care providers. The provider is responsible for determining treatment parameters, making changes in the treatment regimen, and determining the timing of follow-up visits. Between scheduled office encounters, contact between patient and provider is patient initiated. The provider may use clinic nurses to follow-up on problematic patients or patients with abnormal results. In essence, this group receives the current standard of care at the study clinics.

### Content of individual treatment sessions for the TIDES group

#### Session 1

After enrollment, randomization, and completing the baseline assessment, each participant in the TIDES group comes in for a face-to-face meeting with the NCMs or DEs. The primary purpose of the face-to-face visit is to establish rapport and give participants an opportunity to meet their NCM or DE. In addition, during this visit, the NCM or DE goes over the study in detail, obtains patient contact information, primary and alternate telephone numbers, and establishes guidelines for follow-up calls. In addition, participants receive information specific to the group as described below.

#### Sessions 2–13

Participants randomized to the TIDES group receive the culturally tailored education booklet that was developed as part of Racial and Ethnic Approaches to Community Health 2010 titled “Your Guide to Sugar Diabetes”. They are trained on how to use the five patient activation questions during clinic visits. They are also given a patient empowerment package and trained on how to use the materials in a diabetes care package. In addition, the NCM/DE goes over the principles of behavioral skills training, asks the participant to choose the first target behavior, and assists the participant in developing an action plan.

The intervention group receives weekly telephone-delivered diabetes knowledge/information, patient activation, patient empowerment, and behavioral skills training delivered via telephone. Telephone sessions (2–13) for the intervention group are delivered weekly for 12 weeks and last for 30 minutes. Participants randomized to this group participate in discussions on diabetes-related topics and receive motivation/skills training in self-blood sugar monitoring, medication adherence, diet, and physical activity. During the final week of discussion, a review of the major points of each session covered previously is conducted.

#### Sessions 14–15

Treatment group-specific booster sessions for the TIDES group are delivered by telephone at 6 and 9 months post-randomization. A review of the major points of each session covered previously is conducted during the booster sessions.

### Study instruments and data collection schedule

**Figure 4:**
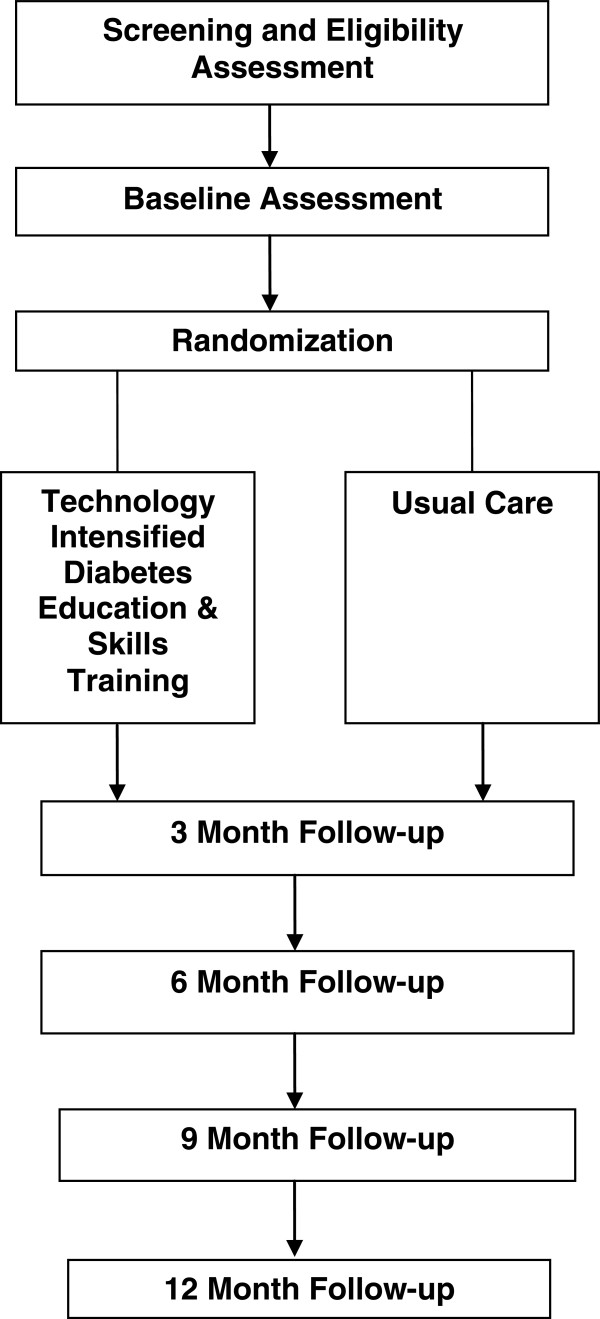
**Design and study flow.**

See Figure 4 and Tables 1, 2, 3 for the study design and study flow, data collection schedule, data collection measures, and data collection instruments, respectively.

**Table 1:** **Data collection schedule**

Questionnaires and measurements	Screening visit	Baseline visit	3-month visit	6-month visit	9-month visit	12-month visit
**Primary outcome measure**						
Glycosylated hemoglobin A1c	X		X	X	X	X
**Secondary outcome measures**						
Blood pressure		X	X	X	X	X
Quality of life (12-item Short-Form Health Survey; SF-12)		X	X	X	X	X
Resource utilization/cost		X	X	X	X	X
**Process measures**						
Diabetes knowledge questionnaire		X	X	X	X	X
Knowledge about diabetes (The Diabetes Study of Northern California survey; DISTANCE)		X	X	X	X	X
Diabetes education (Behavioral Risk Factor Surveillance System; BRFSS)		X	X	X	X	X
Diabetes empowerment scale		X	X	X	X	X
Behavioral skills (Summary of Diabetes Self-Care Activities; SDSCA)		X	X	X	X	X
Medication adherence		X	X	X	X	X
Diabetes fatalism		X	X	X	X	X
**Covariates/self-report measures**						
Patient demographics	X					
Social support		X	X			X
Health literacy		X	X			X
Depression (Patient Health Questionnaire-9; PHQ-9)		X	X			X
Diabetes distress (Diabetes Distress Scale; DDS-2)		X	X			X
Delayed discounting (Quick Delay Questionnaire; QDQ)		X	X			X
Medical comorbidity (chronic health conditions, BRFSS)		X	X			X

**Table 2:** **Data collection measures**

Outcome	Test	Measurement
Primary outcome measure	Glycosylated hemoglobin A1c	Blood specimens will be obtained at baseline, and at 3, 6, 9, and 12 months.
Secondary outcome measures	Blood pressure measurements	Blood pressure readings will be obtained at baseline and at 3, 6, 9, and 12 months by a trained research assistant using automated blood pressure monitors (OMRON IntelliSense™ HEM-907XL) with the patient seated comfortably for 5 minutes prior to the measurements.
	Quality of life	The 12-item Short Form Health Survey (SF-12) [16] is a valid and reliable instrument to measure functional status and will be used to assess quality of life at baseline and at 3, 6, 9, and 12 months. The SF-12 items reproduce at least 90% of the variance in Physical Component Summary-36 and Mental Component Summary-36 scores.
	Resource utilization and cost	The perspective of cost will be that of the payer. Previously validated questions on resource utilization will be administered as part of the baseline and at 3, 6, 9, and 12 months.

**Table 3:** **Data collection instruments**

Measure	Data collected	Method
Process and behavioral measures	Information	This will be measured by the 24-item Diabetes Knowledge Questionnaire (DKQ) [17].
	Knowledge about diabetes	Previously validated items from the Diabetes Study of Northern California (DISTANCE) survey [18] will be used to capture knowledge about diabetes.
	Diabetes education	Three previously validated items from the Behavioral Risk Factor Surveillance System (BRFSS) [19] will be used to capture the patients’ confidence in diabetes self-management.
	Motivation	This will be measured by the eight-item Diabetes Empowerment Scale-Short Form (DES-SF) [20].
	Self-efficacy	This will be measured by the Perceived Diabetes Self-Management Scale (PDSMS) [21].
	Behavioral skills	This will be assessed with the Summary of Diabetes Self-Care Activities (SDSCA) scale [22].
	Medication adherence	This will be measured with the new eight-item self-report Morisky Medication Adherence Scale (MMAS) [23].
	Diabetes fatalism	This will be measured with the 12-item Diabetes Fatalism Scales (DFS) [24], a valid and reliable measure of diabetes fatalism that is associated with self-care problems, poor glycemic control, and decreased quality of life.
Covariates	Demographics	Previously validated items from the 2002 National Health Interview Survey [25] will be used to capture age, gender, race/ethnicity, marital status, household income, and health insurance.
	Social support	The Medical Outcomes Study (MOS) Social Support Survey [26] will be used to measure social support.
	Health literacy	The abbreviated version of the Test of Functional Health Literacy in Adults (S-TOFHLA) [27] is designed to rapidly screen patients for potential health literacy problems.
	Depression	The Patient Health Questionnaire-9 (PHQ-9) is a brief questionnaire that scores each of the nine Diagnostic and Statistical Manual of Mental Disorders (4th revision) criteria for depression [28].
	Diabetes distress	The Diabetes Distress Scale (DDS) is a two-item scale that measures patient concerns about disease management, support, and emotional burden [29].
	Delayed discounting	The Quick Delay Questionnaire (QDQ) Discounting Module is a 10-item survey used to help researchers understand how the amount of monetary gain and timing are associated with treatment adherence and clinical outcomes among individuals with type 2 diabetes [30].
	Medical comorbidity	The patient's history of medical comorbidity will be documented using chronic health conditions (previously validated items from the BRFSS) [31].

### Primary outcome measure

The primary outcome is HbA1c level at 12 months of follow-up.

### Sample size determination and power analysis

For the primary and secondary efficacy outcomes, HbA1c (primary outcome) and BP, QoL, and resource utilization/cost (secondary outcomes), with 80 subjects randomized to each of the two groups, we have 85% power to detect at least a 0.4 standardized effect size (difference in comparison group means in standard deviation (SD) units) for continuous outcome measures. This calculation assumes that: the primary and secondary efficacy outcomes are measured at five time points (baseline, 3, 6, 9, and 12 months); the intra-class correlation for repeated observations is no greater than 0.7; the level of significance (α) = 0.05, two-tailed test. Assuming HbA1c, systolic BP, and diastolic BP measurements have standard deviations of approximately 2.0. 20.6, and 9.6, respectively, based on our pilot studies, the study has 85% power to detect a difference of 0.8 percentage points (raw units) in HbA1c, 8.2 mmHg in systolic BP, and 3.8 mmHg in diastolic BP between the two comparison groups. Analyses for continuous QoL, process and behavioral variables also have 85% power to detect a standardized effect size of 0.4 SD. To account for the “fraction of missing” information that must be imputed in the intent-to-treat (ITT) sample and the dilution effect of ITT analyses [30], the sample size is inflated by 20% to achieve a final ITT sample size of 100 subjects randomized to each treatment group (N = 200).

### Data analysis

#### Primary hypotheses

Among AAs with poorly controlled T2DM, patients randomized to the TIDES intervention will have significantly greater reduction in HbA1c at 12 months of follow-up compared to usual care.

The ITT sample, comprising all randomized patients, will be used for the primary analyses [14]. The per protocol/completer sample will comprise participants who were compliant with protocol requirements and for whom all required measurements over 12 months of follow-up have been made. Analyses will be carried out separately for ITT and per protocol samples to test sensitivity of conclusions to drop-outs/non-adherence. If differences are present between the per protocol and ITT analysis sets, the characteristics of the two analysis populations will be examined to aid in explaining any discrepancies.

For primary analyses of longitudinal data, we will employ longitudinal data methods (mixed effects models) which allow for missing data [32]. In addition, we will model the dichotomous outcome, missing/not missing end-of-study score, using logistic regression to determine if missingness is related to intervention group and/or initial level of the given outcome variable (baseline score). Missing covariate data will be imputed using the multiple imputation method of Little and Rubin [33] if needed.

We specify *a priori* the primary (HbA1c) and secondary (BP and QoL) measures corresponding to *a priori* hypotheses, and therefore maintain the significance level (alpha) at 0.05 for these analyses, with no correction for multiple outcomes. Other measures (for example, behavioral outcomes, process measures) are considered secondary/exploratory. We will carry out both unadjusted and multiplicity-adjusted (using a Bonferroni-type correction) analyses involving these variables, and will report both unadjusted and adjusted *P*-values in the event that conclusions differ based on adjustment.

## Discussion

The proposed study provides a unique opportunity to address existing gaps in the literature by testing a combined telephone-delivered, diabetes knowledge/information and motivation/behavioral skills training intervention in AAs with poorly controlled T2DM. The combined diabetes knowledge/information and motivation/behavioral skills intervention focuses on four key behaviors (physical activity, diet, medication adherence, and self-monitoring of blood glucose), maintains adequate dose and intensity of the interventions by delivering the interventions weekly for 12 weeks, and allow patients to choose one behavior to address every 3 weeks. The findings of this study, if successful, will lead to the implementation of this feasible, evidence-based intervention for high-risk minority patients with T2DM. The study findings will also provide new information on how to improve quality of care for diabetes in ethnic minorities and reduce the disproportionate burden of diabetes complications and deaths in ethnic minority groups with T2DM.

## Trial status

The study was funded in May 2013. Study recruitment began in December 2013, with all follow-up assessments associated with the study expected to be completed by April 2017. As of 30 October 2014, 295 patients have been scheduled, 171 enrolled, and 106 randomized. Of the number randomized, 24 have completed the 12-week phone interventions, and 66 have completed the 3-month follow-up assessment, 65 have completed the 6-month follow-up assessment, and 23 have completed the 9-month follow-up assessment.

## Abbreviations

AA: African American
BP: blood pressure
DE: diabetes educator
HbA1c: glycosylated hemoglobin A1c
IRB: Institutional Review Board
ITT: intent-to-treat
NCM: nurse case manager
QoL: quality of life
T2DM: type 2 diabetes mellitus
TIDES: technology-intensified diabetes education/skills training.
